# Supplementary figures and images for: The Effect of Synthetic Polyamine BPA-C8 on the Fertilization Process of Intact and Denuded Sea Urchin Eggs
Source: Cells. 2024 Sep 2;13(17):1477. doi: 10.3390/cells13171477 (PMC11394060; doi:10.3390/cells13171477)

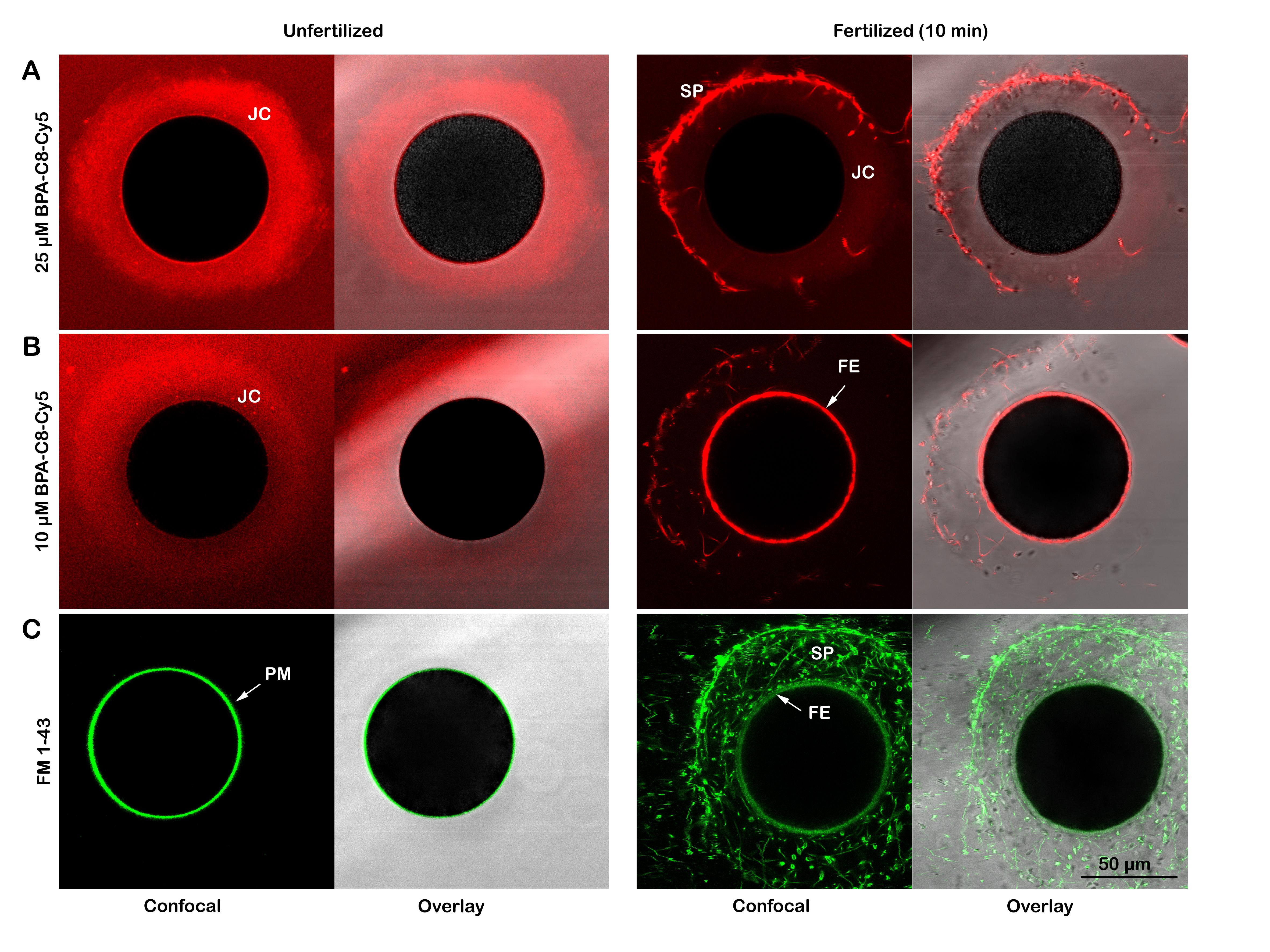

Supplement: Supplementary file 1 [file cells-13-01477-s001.zip › Supplementary F1.jpg]

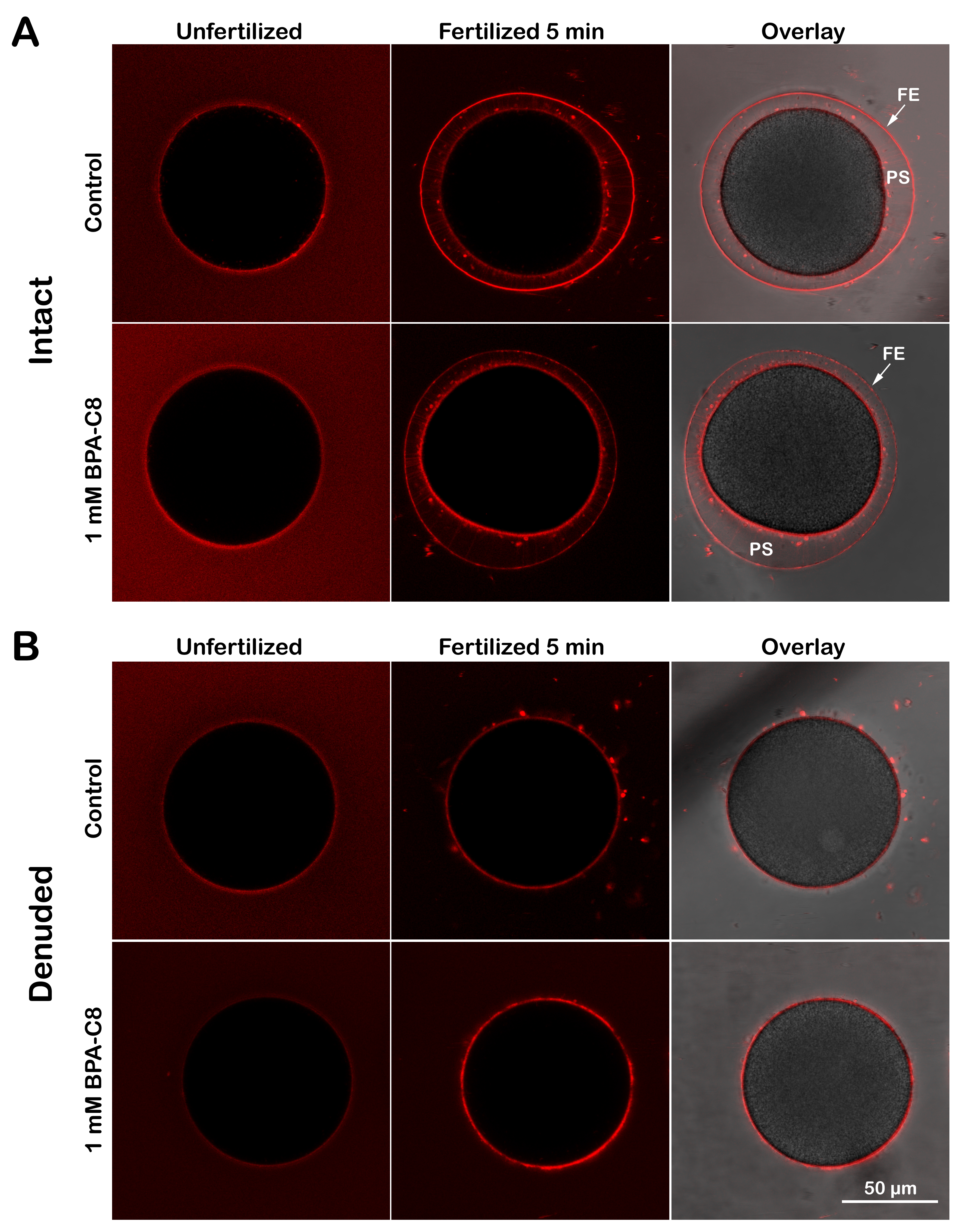

Supplement: Supplementary file 1 [file cells-13-01477-s001.zip › Supplementary F2.jpg]
